# Methods for high-throughput MethylCap-Seq data analysis

**DOI:** 10.1186/1471-2164-13-S6-S14

**Published:** 2012-10-26

**Authors:** Benjamin AT Rodriguez, David Frankhouser, Mark Murphy, Michael Trimarchi, Hok-Hei Tam, John Curfman, Rita Huang, Michael WY Chan, Hung-Cheng Lai, Deval Parikh, Bryan Ball, Sebastian Schwind, William Blum, Guido Marcucci, Pearlly Yan, Ralf Bundschuh

**Affiliations:** 1 The Ohio State University Comprehensive Cancer Center, Columbus, Ohio, USA; 2 Graduate Institute of Medical Sciences, Department of Obstetrics and Gynecology, Tri-Service General Hospital, National Defense Medical Center, Taipei, Taiwan; 3 Department of Life Science, National Chung Cheng University, Min-Hsiung, Chia-Yi, Taiwan; 4 Departments of Physics and Biochemistry, Center for RNA Biology, The Ohio State University, Columbus, Ohio, USA

## Abstract

**Background:**

Advances in whole genome profiling have revolutionized the cancer research field, but at the same time have raised new bioinformatics challenges. For next generation sequencing (NGS), these include data storage, computational costs, sequence processing and alignment, delineating appropriate statistical measures, and data visualization. Currently there is a lack of workflows for efficient analysis of large, MethylCap-seq datasets containing multiple sample groups.

**Methods:**

The NGS application MethylCap-seq involves the *in vitro *capture of methylated DNA and subsequent analysis of enriched fragments by massively parallel sequencing. The workflow we describe performs MethylCap-seq experimental Quality Control (QC), sequence file processing and alignment, differential methylation analysis of multiple biological groups, hierarchical clustering, assessment of genome-wide methylation patterns, and preparation of files for data visualization.

**Results:**

Here, we present a scalable, flexible workflow for MethylCap-seq QC, secondary data analysis, tertiary analysis of multiple experimental groups, and data visualization. We demonstrate the experimental QC procedure with results from a large ovarian cancer study dataset and propose parameters which can identify problematic experiments. Promoter methylation profiling and hierarchical clustering analyses are demonstrated for four groups of acute myeloid leukemia (AML) patients. We propose a Global Methylation Indicator (GMI) function to assess genome-wide changes in methylation patterns between experimental groups. We also show how the workflow facilitates data visualization in a web browser with the application Anno-J.

**Conclusions:**

This workflow and its suite of features will assist biologists in conducting methylation profiling projects and facilitate meaningful biological interpretation.

## Background

Advances in whole genome profiling technologies have revolutionized the field of cancer research. These technologies have facilitated the discovery of potential biomarkers for disease development and progression as well as our understanding of the complex, underlying molecular mechanisms that lead to cancer. Reduction in costs have spurred the adoption of next generation sequencing (NGS) platforms which offer greater resolution and sensitivity compared to traditional microarray profiling [1]. At the same time, NGS raises new bioinformatics challenges, both practical (e.g. data storage, computational costs) and theoretical (e.g. defining appropriate statistical measures).

A promising application of NGS is the whole-genome profiling of epigenetic modifications, including DNA methylation. The addition of methyl groups to the 5' carbon position of cytosine bases is a major mechanism of epigenetic regulation which participates in reorganizing chromatin structure and silencing gene expression [2], Epigenetic alterations, such as tumor suppressor gene hypermethylation and oncogene hypomethylation, are hallmarks of cancer and play a pivotal role in tumorgenesis and disease progression [3,4].

The DNA methylation profiling approach used in our lab, MethylCap-seq involves the in vitro capture of methylated DNA with the high affinity methyl-CpG binding domain of human MBD2 protein and subsequent analysis of enriched fragments by massively parallel sequencing [5-8]. Benchmarking has shown MethylCap-seq is more effective at interrogating CpG islands than antibody-based methylated DNA immunoprecipitation sequencing (MeDIP-seq) [9]. While optimizing this experimental technique, we recognized two potential issues affecting subsequent data analysis. First, unsuccessful or incomplete capture reactions can result in the sequencing of non-methylated DNA fragments, leading to inconsistencies in or the absence of methylation enrichment in a sample. Second, poor sequencing library complexity and CpG coverage limit the statistical power to call differential methylation, and ultimately the reproducibility of the dataset. Conventional sequencing analysis pipelines often do not include assay-dependent quality control assessments. Spurious samples reduce analytical power and lead to excess "noise" in downstream analyses.

The challenges to data analysis are real. The numerous options for file processing and genome alignment mean any particular strategy requires extensive troubleshooting and optimization. Large file sizes make data visualization exceedingly difficult without the use of expensive commercial software packages or system resource-intensive publicly available programs. In more practical terms, MethylCap-seq projects, in particular, would greatly benefit from the ability to receive rapid feedback of overall experimental quality. There is also a lack of workflows for efficient analysis of large, MethylCap-seq datasets containing multiple sample groups. To address these pertinent issues, we have developed a scalable, flexible workflow for MethylCap-seq Quality Control and secondary data analysis which facilitates tertiary analysis of multiple experimental groups and data visualization.

The automated MethylCap-seq workflow has been developed over the course of 200 sequencing runs. The workflow is scalable in terms of handling studies of disparate sample sizes. It is flexible in that unique experimental considerations (genome alignment, read bin sizes, test statistics) can be addressed by simple modification of several operational parameters independent of the scripts responsible for automating the workflow. Automation is imperative because of the large number of intermediate steps and temporary files required. The workflow incorporates proven, existing tools where applicable: e.g., raw read processing, the short read aligner, the R environment and third party libraries. It further takes advantage of high performance computing systems for parallel batch job submissions. This feature is important for scalability and computational feasibility. Data visualization is supported by Anno-J, a genome annotation visualization program and web service viewport.

## Methods

### Patient samples

A total of 71 ovarian cancer samples, 6 adjacent normal tissues as well as 20 tissues collected from patients during surgery for benign gynaecological disease were obtained from Triservice General Hospital, Taipei, Taiwan. All studies involving human ovarian cancer samples were approved by the Institutional Review Boards of Triservice General Hospital and National Defense Medical Center.

### Methylated-DNA capture (MethylCap-seq)

Enrichment of methylated DNA was performed with the Methylminer kit (Invitrogen) according to the manufacturer's protocol. Briefly, one microgram of sonicated DNA was incubated at room temperature on a rotator mixer in a solution containing 3.5 micrograms of MBD-Biotin Protein coupled to M-280 Streptavidin Dynabeads. Non-captured DNA was removed by collecting beads on a magnet and washing three times with Bind/Wash Buffer. Enriched, methylated DNA was eluted from the bead complex with 1 M NaCl and purified by ethanol precipitation. Library generation and 36-bp single-ended sequencing were performed on the Illumina Genome Analyzer IIx according to the manufacturer's standard protocol. Each sample was sequenced on a single lane, for a total of 97 lanes. Additional data sets are presented for demonstration purposes only.

### MethylCap-seq experimental QC

**Figure 1:**
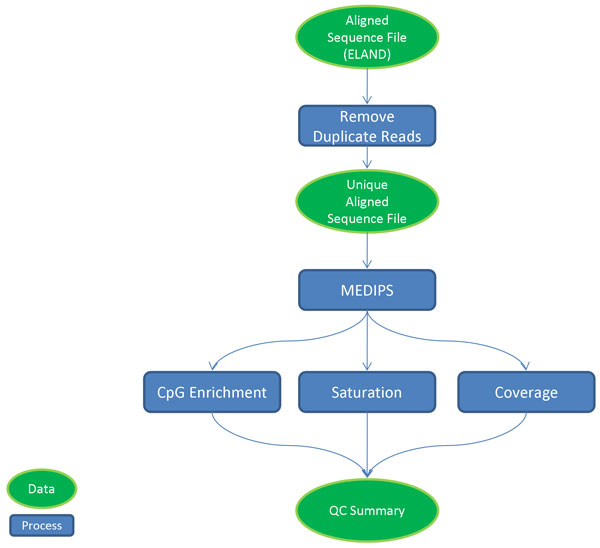
**Diagram of MethylCap-seq QC workflow**. Arrows indicate order of operation. Green ovals, Data; Blue Rectangles, Process.

The quality control module identifies technical problems in the sequencing data and flags potentially spurious samples. The module is based on MEDIPs [10], an enrichment-based DNA methylation analysis package, and provides rapid feedback to investigators regarding dataset quality, facilitating protocol optimization prior to committing resources to a larger scale sequencing project. Figure 1 illustrates the QC automated workflow. For each aligned sequencing file (e.g., the default output of Illumina's CASAVA pipeline), duplicate reads are removed (a correction for potential PCR artifacts), and a stripped, uniquely aligned sequence BED file is loaded into an R workspace for processing by the MEDIPS library. Three functions are performed on the data: Saturation analysis, CpG enrichment calculation, and CpG coverage analysis. Saturation analysis performs a Pearson correlation coefficient estimation of sequencing library complexity and potential reproducibility. CpG enrichment calculation consists of the relative CpG dinucleotide frequency interrogated by aligned sequence reads divided by the relative frequency of CpG dinucleotides in the reference genome. CpG coverage rate (5×) is the fraction of CpG dinucleotides in the reference genome sequenced at least five times. The automated workflow produces a QC summary file containing the MEDIPs results and sequencing output metrics from the Illumina CASAVA pipeline. The QC module utilizes the parallel processing capability of a supercomputing environment to greatly decrease the time required for analysis.

### Sequence file processing and alignment

**Figure 2:**
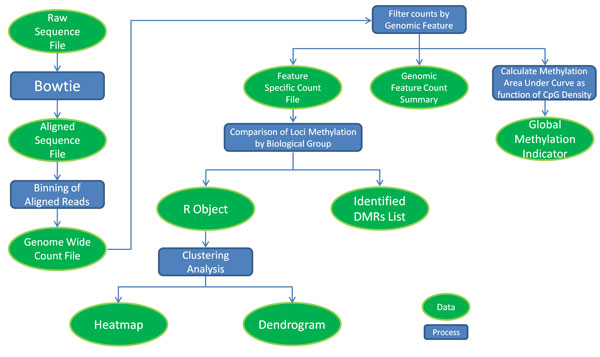
**Diagram of global methylation analysis workflow**. Arrows indicate order of operation and break at decision trees. Green ovals, Data; Blue Rectangles, Process.

The ability to use multiple custom sequence alignment policies facilitates analysis of various genomic regions and features. Bowtie, a short read aligner, provides many alignment policies and options that allow a great deal of customization of the alignment output[11]. While our focus and workflow centers on reporting uniquely aligned reads, alternative alignment options are used for more customized data analysis. The qseq files are preprocessed for a uniquely aligned Bowtie output by being converted to FASTA format. The converted file is then aligned by Bowtie with options that optimize for uniquely aligned reads and output in SAM format. Post processing uses various SAMtools [12] commands to convert the alignment to BAM format and remove all duplicate reads from the alignment before converting back to a final SAM alignment. The workflow, illustrated in Figure 2, is concisely handled by a single script which passes each intermediate stage of the alignment process to the subsequent stage and outputs a single SAM alignment file and a report of the number of reads that were aligned and those which were counted as duplicates. Speed is increased by Bowtie's multithreading options and by performing the alignment in a supercomputing environment. To achieve alternative alignments, Bowtie options can be changed, and different genomic sequences or subsets of genomic sequences may be used for alignment. With minor modification our workflow can be run with other short read aligners that generate SAM files as output.

### Global methylation analysis workflow

The methylation analysis workflow is outlined in Figure 2. Chromosomal coordinates of sequence reads are parsed from the final alignment output, then counted using a specified bin size and read extension length (reflecting average fragment size) in order to generate a binary file containing bin counts and scaled count values (reads per million - rpms). The bin size determines the computational resolution of the analysis. We find that a bin size of 500 bp provides sufficient analysis resolution while smoothing the data statistically. The binary counts file is next interrogated by genomic feature (e.g., CpG islands, CpG shores, Refseq genes) to generate feature-specific count files. The workflow is compatible with custom feature files listing genomic loci of interest in BED format. In addition, aggregate read count summaries can be compiled for each type of genomic feature. Our approach of binning aligned reads, scaling read count values, and/or generating genomic feature-specific count files could prove applicable to other enrichment-based sequencing methods. For instance, the process responsible for filtering counts by genomic features might be modified to accept ChIP-seq peak calling values.

Once the samples are binned and genomic features are extracted, they are grouped based on biological factors, such as known genotype difference, and statistical tests are performed to discern if there are significant differences in methylation counts among predefined groups of samples. One locus from a genomic feature in one group is tested against the same locus in the other group for all loci in that genomic feature. The statistical test used is dependent on the number of groupings. For two groups a Wilcoxon rank-sum test is employed to test the distribution of methylation counts for each locus across the two groups. We then select significant differentially methylated loci by applying a multiple test corrected p-value cutoff (False Discovery Rate, FDR). Similarly for groupings of more than two biological factors, the Kruskal-Wallis test is employed. Statistical testing of genomic features is a custom workflow implemented in R which utilizes the predefined Wilcoxon and Kruskal-Wallis test functions. The output of the workflow is a list of loci from each genomic feature that passes significance testing. Boxplots are also created for the list of significant features for visualization of their differential methylation.

To assess genome-wide changes in methylation patterns between experimental groups, we calculate a Global Methylation Indicator (GMI) for each individual sample in different groups. First, the sample's methylation distribution, an average rpm for each CpG content classification, is determined. The distribution is obtained as follows: each 500 base bin is classified by the CpG content (# of CG base sequences, counting any CG base sequences straddling the end of the bin and the beginning of the next) within the 500 bases it covers. Then within each CpG content classification, the average rpm per bin is calculated by summing the rpms and dividing by the number of bins. The difference between two groups is calculated by using a t test on the estimation of the area under the curve for each individual sample.

### Clustering

To identify novel classifications of samples independently of predefined biological factors, unsupervised clustering of the data can be implemented. Clustering of the data is a workflow that takes a data matrix of the samples and the rpm value of each locus for a given genomic feature. The workflow is implemented in R and utilizes various R libraries for matrix manipulation, flashClust, and pvclust for unsupervised clustering. Adjusted p-values are obtained via multiscale bootstrap resampling of the data. Many combinations of correlation calculations and clustering methods can be implemented. Our clustering workflow uses the Pearson correlation distance measure. It takes as input the "raw" rpm data values or rescaled rpms, depending on the features of interest in the dataset. Rescaling the rpms involves dividing the rpms of each locus by the average rpm for that locus. This allows Pearson correlation to evenly weight both the low and high rpm values. Using the raw rpms causes Pearson correlation to more heavily weight the high rpms. The default clustering method of the workflow is that of McQuitty, but R provides any number of additional choices. Our workflow also implements data selection criteria that enforce a minimum coefficient of variation (CV) threshold in combination with minimum average rpm threshold for each locus. In tandem with the dendrograms, heatmaps are also produced to help visualize the relationship between the clustering sample members. This entire workflow, including all combinations of selection criteria and all genomic features of interest, is completed in a single script.

Because we produce a variety of dendrograms through the use of various genomic features and loci selection criteria, it is useful to see if the membership of a significant group is conserved throughout the dendrograms that were created using other genomic features and even within genomic features analyzed with varying selection criteria. To easily visualize the location of a certain sample group's membership in other dendrograms, we use different colors to track the membership of that group through alternative dendrograms that are produced for different genomic features and selection criteria. Tracking the membership of a group is accomplished by supplying the membership of that group to a color function that can be applied to subsequent dendrograms through the dendrapply function in R.

### Data visualization

In our workflow, we have incorporated Anno-J, a REST-based Web 2.0 application for the visualization of deep sequencing information and other genomic annotations [13]. Anno-J is capable of streaming all necessary applets and scripts to the user, providing immediate and installation-less viewing within a user's web browser. This facilitates the fast, real-time and interactive visualization of multiple data sets by users with access to any server hosting Anno-J. Data visualization within Anno-J uses tracks, discrete rows of graphs, each of which corresponds to a particular set of data. Our workflow incorporates a number of custom scripts which allow quick conversion of binary and raw text read counts and SAM files to various Anno-J track formats, including standard mask and read tracks. These scripts extract from read count files the location and rpm, and from SAM files the location, sequence count and strand identifier, and generate Anno-J read track format files. With minor modification, the scripts could be used to generate data tracks compatible with the UCSC Genome Browser. For the Anno-J experiment tracks, a scheduled service loads any new files from a shared folder into our database using a prescribed data format. Each track is assigned a unique identifier and properties for experiment type (e.g., methylation, small RNA) and track type (e.g., read, mask). The Anno-J web application will configure the browser with specified tracks based on these properties. The browser calls web services which return formatted data for each track, filtered by the currently viewable portion of the chromosome.

Additionally, we have incorporated a custom algorithm which allows conversion of binary and raw text read counts files to a custom discretized methylation heatmap track format. The heatmap track format modifies constraints and features of the Anno-J mask track format to allow generation of individual rows of heatmap data. Discretized methylation heatmap tracks are created by percentile ranking binned rpm values from binary or raw text read counts files, and then assigning color gradient based upon rank. Generation of the final discretized heatmap is a matter of stacking multiple heatmap tracks together.

## Results and discussion

### Experimental quality control

The automated MethylCap-seq workflow has been developed over the course of 200 sequencing runs. It has been applied to human solid tumors (e.g., breast, ovarian, endometrial, and hepatocellular carcinoma) and blood cancers (e.g., acute myeloid leukemia, chronic lymphocytic leukemia) as well a number of mouse cancer models. Though untested in that context, our analysis workflow should be applicable to other enrichment-based methylation assays such as MeDIP-seq studies.

**Figure 3:**
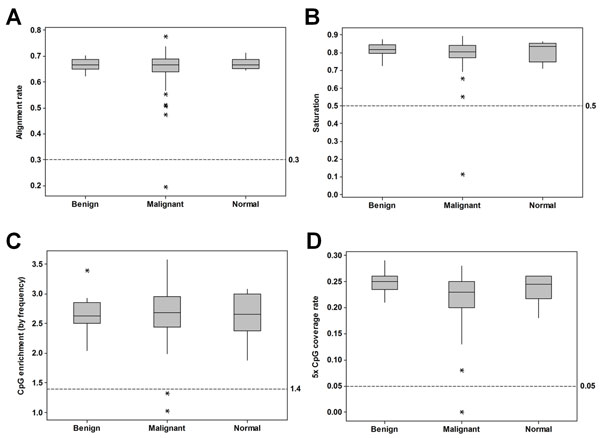
**QC analysis of a large sample cohort**. The QC values associated with a large ovarian cancer study dataset are presented in box plots according to sample type (normal = 6, benign = 20, malignant = 71). In each plot, threshold cutoff values for sample exclusion are indicated by hatched horizontal reference lines. A, Box plot of MethylCap-seq unique read alignment rate. The fraction of pass-filter reads which align uniquely to the human reference genome. B, Box plot of Saturation analysis, a Pearson correlation coefficient estimation of sequencing library complexity and potential reproducibility. C, Box plot of CpG enrichment, the relative CpG dinucleotide frequency interrogated by aligned sequence reads divided by the relative frequency of CpG dinucleotides in the reference genome. D, Box plot of CpG coverage rate (5×), the fraction of CpG dinucleotides in the reference genome sequenced at least five times.

The QC workflow runs immediately after the sequencing experiment has been transferred from the Illumina Real Time Analysis (RTA) pipeline. It calls several functions of the R package MEDIPS [10] and reads the Illumina RTA run summary output. From our QC workflow, we have found the following parameters considered collectively can flag problematic samples: CpG enrichment, saturation, CpG coverage, and alignment rate. Even valid samples occasionally fail a single parameter; thus, we typically exclude those which fail two or more parameters. QC results from a large ovarian cancer study dataset (97 patient samples) are shown in Figure 3A-D. 2 of 97 lanes of data were excluded from further analysis. In a second large cancer dataset (105 patients) where the majority of samples were sequenced on multiple lanes (207 in total), 43 (20.8%) qualified for exclusion. Sequencing of new libraries generated for 12 samples with prior insufficient aligned reads all failed the QC again, demonstrating how sample intrinsic factors (such as DNA quality or integrity) dramatically impact the quality of MethylCap sequencing data. CpG enrichment, the frequency of CpG dinucleotides observed in the sequenced sample compared to the expected frequency in the reference genome, is likely the most significant QC parameter because it can indicate failure of the initial methylation-capture step. As MethylCap-seq is an enrichment-based approach, identifying failures in enrichment is imperative. We observed an average CpG enrichment value of 2.70 ± 0.35 in the ovarian cancer dataset. In general, enrichment values range from 2 - 3.5 and show similar distributions in samples from normal and malignant tissues as shown for the ovarian cancer dataset (Figure 3C). Enrichment values from input samples (non-captured DNA) are less than or near 1. We routinely exclude samples with CpG enrichment values less than 1.4. Such samples usually have low saturation values as well (less than 0.5), a measure of the statistical reproducibility of the dataset, suggesting that the methylation calls would be difficult to reproduce if the library was resequenced.

### Differential methylation analysis of multiple sample groups

**Figure 4:**
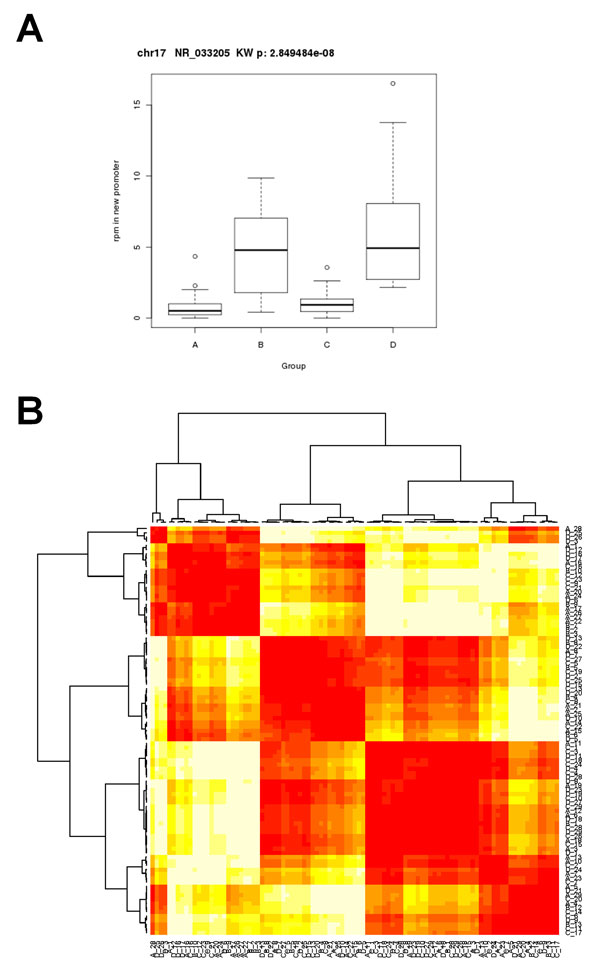
**Methylation analysis of multiple sample groups**. A, Boxplot of noncoding RNA NR_033202 promoter methylation in four groups of AML patients. Multiple-testing corrected non-parametric Kruskal-Wallis analysis of variance p-value is shown. B, Hierarchical clustering dendrogramand heatmap of methylation in gene promoters among four groups of AML patients. The feature threshold criteria were avg rpm > 10 and CV > 5. The full dendrogram generated in R with the pvclust package is included in Additional file 1.

**Figure 5:**
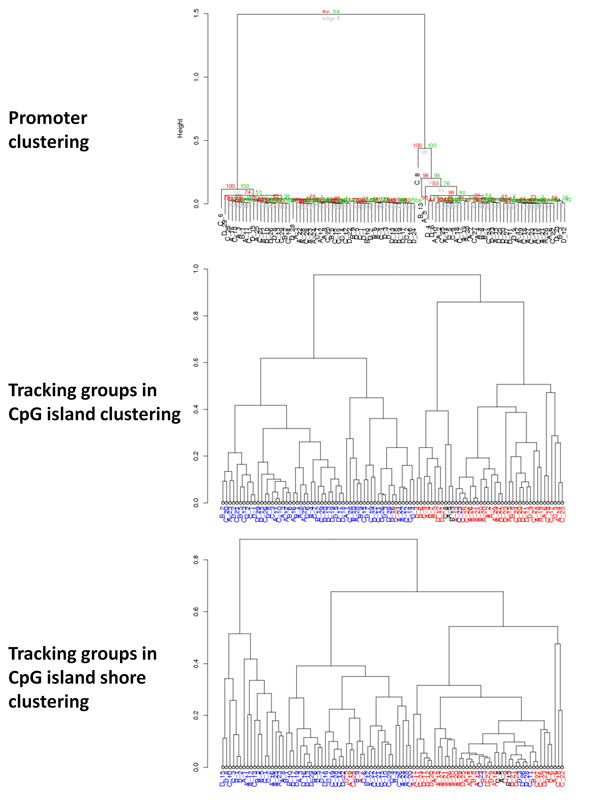
**Tracking groups in hierarchical clustering**. The top panel shows the initial clustering dendrogram of methylation in gene promoters among four groups of AML patients (feature threshold criteria: average rpm > 0, CV > 3). Membership in the two largest clusters is then tracked in subsequent dendrograms by blue (left cluster) and red (right cluster) sample labels. Black labels indicate samples not present in the two largest clusters. Middle panel, clustering dendrogram of CpG island methylation (feature threshold criteria: average rpm > 0, CV > 3). Bottom panel, clustering dendrogram of methylation in CpG island shores (feature threshold criteria: average rpm > 0, CV > 3).

Current strategies for enrichment-based sequencing differential methylation analysis have been limited to individual pair-wise sample comparisons such as tumor versus normal [9] or comparisons of multiple samples (in pair-wise fashion) to a common normal reference sample [14]. Thus a salient feature of our workflow is the ability to compare methylation profiles of multiple samples in two or more biological groups. Significance testing is performed in R with the non-parametric Wilcoxon (two groups) or Kruskal-Wallis (> two groups) tests. An example of promoter methylation profiling analysis for four AML patient groups is shown in Figure 4A-B. Results of individual features can be visualized by whisker plots as in Figure 4A which shows differential methylation of the NR_033205 transcript promoter. The workflow performs unsupervised clustering in order to identify novel classifications of samples (Figure 4B, Additional file 1). In methylation profile clustering analysis, data selection criteria are enforced in order to pare down the number of loci being used for clustering within each genomic feature. The rationale for this approach is that it allows the clustering to be performed on only the loci with the largest differences among samples; the minimum rpm value for each locus removes loci that were not pulled down well during sequencing and thus are expected to be rather noisy. Hierarchical clustering of promoter regions passing threshold criteria (avg rpm > 10 and CV > 5) reveals four distinct patient groupings (Figure 4B, Additional file 1). Combinations of the selection criteria produce many different dendrograms of the data for evaluation and serve as a method for exploration of novel differentially methylated loci that may contribute to biological factors. To determine if membership of a significant group is conserved among dendrograms created using other genomic features or within genomic features analyzed with varying selection criteria, we implement a group tracking function as shown in Figure 5. If the membership of a group is conserved as we track it through alternative dendrograms, it is more likely to be biologically significant rather than an artifact of the specific clustering procedure.

### Assessing genome-wide methylation patterns

**Figure 6:**
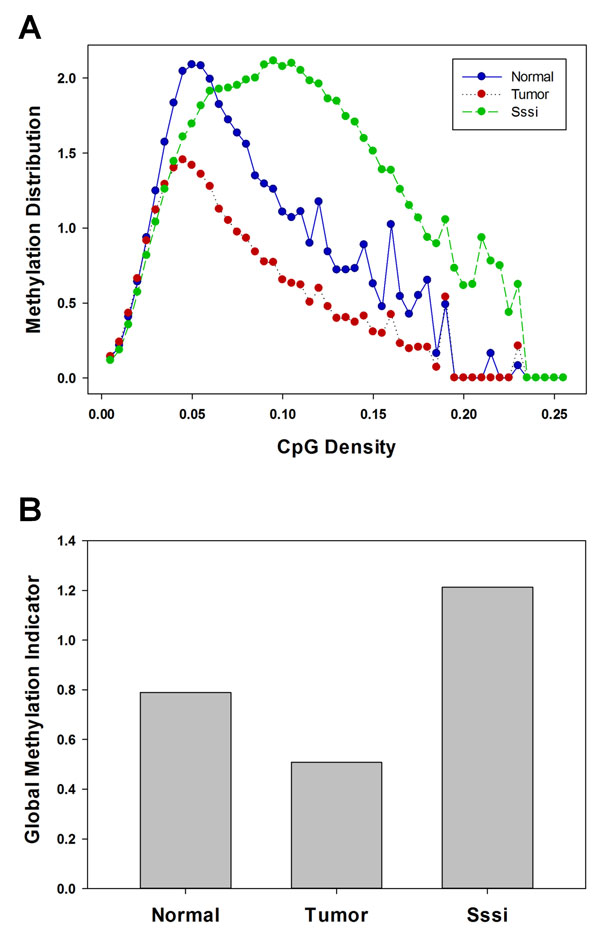
**Assessment of global DNA methylation patterns**. A, Methylation Distribution plot of a normal ovarian tissue (blue circles), an ovarian carcinoma (red circles), and an *in vitro *methylated (Sssi) positive control sample. B, Global Methylation Indicator (GMI) values of the three samples.

To assess genome-wide changes in methylation patterns between experimental groups independent of genomic features, we calculate a Global Methylation Indicator (GMI) for each individual sample in different groups. Comparison of two or more Methylation Distributions provides information regarding the differences in average methylation at various CpG classifications. A representative Methylation Distribution plot presented in Figure 6A shows the distributions of a normal ovarian tissue, an ovarian carcinoma, and an *in vitro *methylated positive control. Likewise, comparing two or more GMIs may provide information regarding gross differences in global methylation. A GMI plot corresponding to the three samples in Figure 6A is provided in Figure 6B.

### MethylCap-seq data visualization

**Figure 7:**
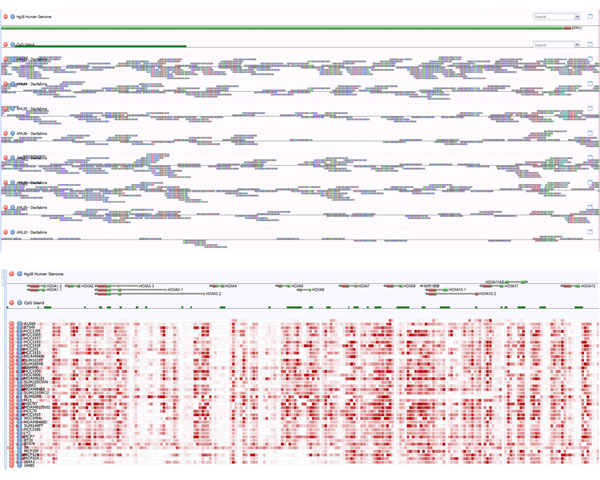
**Data visualization with Anno-J**. Top, Methylation read data at single base resolution. Data depicted are the 5' end of the *EPPK1 *gene (track 1) and associated CpG island (track 2) in eight AML patient samples (tracks 3 - 10). Bottom, methylation heatmap of the *HOXA *gene cluster in 35 breast cancer cell and five normal breast epithelial cell lines (last five rows).

Effective data visualization can bridge the divide between computational and experimental biologists engaged in integrated analysis projects. Visual interpretation of patterns may permit the researcher to observe phenomena which computational analysis do not detect. The data workflow prepares samples for visualization in a web browser with the application Anno-J (Figure 7). In Anno-J, samples are represented as individual data tracks which can traversed, scaled and rearranged interactively by the user in real-time. Individual sequence reads can be visualized at single-base resolution as demonstrated in the top panel of Figure 7 which depicts methylation read data at the *EPPK1 *gene locus in eight AML patient samples. To interact with data at a much broader resolution, we developed a custom methylation heatmap data track. The bottom panel of Figure 7 shows a methylation heatmap of the *HOXA *gene cluster in breast cancer cells (n = 35) and normal breast epithelial cell lines (n = 5).

## Conclusions

In this paper, we presented a scalable, flexible workflow for performing MethylCap-seq Quality Control, secondary data analysis, tertiary analysis of multiple experimental groups, and data visualization in the web service viewport, Anno-J. As the cancer epigenetics field further expands into next generation sequencing, our workflow should assist biologists in conducting methylation profiling projects and facilitate meaningful biological interpretation.

## Competing interests

The authors declare that they have no competing interests.

## Authors' contributions

BR, DF, and MM conceived the computation experiments. JC, RH, and MC performed laboratory experiments. BR, DF, MM, HT, and MT analyzed data. SS and WB provided patient information and clinical interpretation. DP and BB were responsible for web and database development. BR, PY, and RB co-wrote the paper. PY, RB, HL, and GM provided guidance. All authors discussed the results and commented on the manuscript. All authors read and approved the final manuscript.

## Supplementary Material

Additional file 1 **Promoter methylation hierarchical clustering dendrogram**. Click here for file
