# Supplementary material for: Methods for high-throughput MethylCap-Seq data analysis
Source: BMC Genomics. 2012 Oct 26;13(Suppl 6):S14. doi: 10.1186/1471-2164-13-S6-S14 (PMC3481483; doi:10.1186/1471-2164-13-S6-S14)
Supplement: Additional file 1 — Promoter methylation hierarchical clustering dendrogram. [file 1471-2164-13-S6-S14-S1.pdf]

Additional File 1. Promoter methylation hierarchical clustering dendrogram

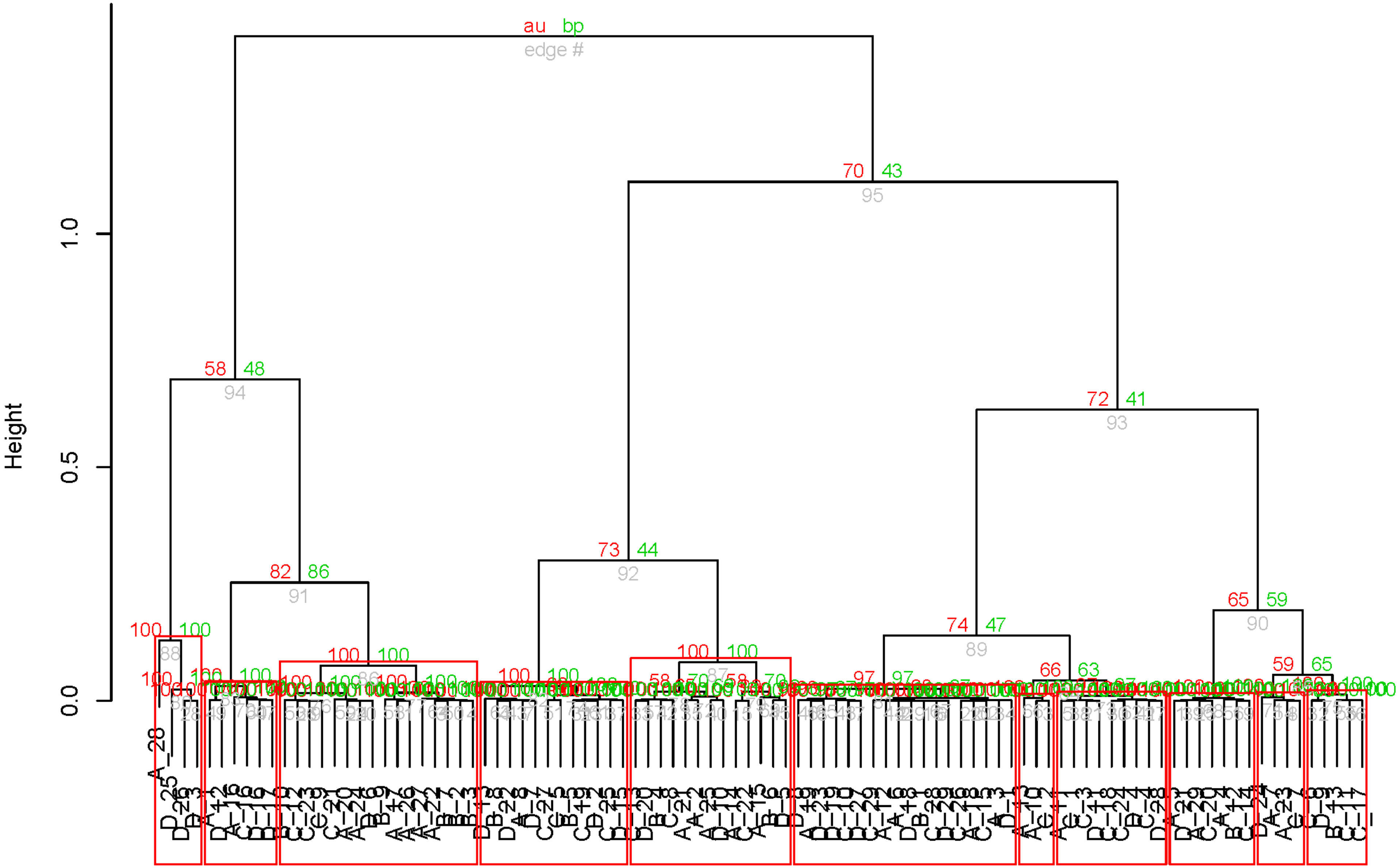

Hierarchical clustering dendrogram of methylation in gene promoters among four groups of AML patients. Clustering was performed with the R package pvclust. The feature threshold criteria were avg rpm > 10 and CV > 5. Values at branches represent multiscale bootstrapping calculated approximately unbiased (AU) p-values (red) and bootstrap p-values (green). Red boxes indicate cluster branches which meet the AU p-value threshold for significance. Cluster labels indicating group membership are shown below the branches.
